# Resilience in carbonate production despite three coral bleaching events in 5 years on an inshore patch reef in the Florida Keys

**DOI:** 10.1007/s00227-018-3354-7

**Published:** 2018-05-08

**Authors:** Derek P. Manzello, Ian C. Enochs, Graham Kolodziej, Renée Carlton, Lauren Valentino

**Affiliations:** 1 Atlantic Oceanographic and Meteorological Laboratories (AOML), NOAA, 4301 Rickenbacker Cswy., Miami, FL 33149 USA; 2 Cooperative Institute for Marine and Atmospheric Studies, Rosenstiel School of Marine and Atmospheric Science, University of Miami, 4600 Rickenbacker Cswy., Miami, FL 33149 USA; 3 Khaled bin Sultan Living Oceans Foundation, Landover, MD USA

## Abstract

The persistence of coral reef frameworks requires that calcium carbonate (CaCO_3_) production by corals and other calcifiers outpaces CaCO_3_ loss via physical, chemical, and biological erosion. Coral bleaching causes declines in CaCO_3_ production, but this varies with bleaching severity and the species impacted. We conducted census-based CaCO_3_ budget surveys using the established *ReefBudget* approach at Cheeca Rocks, an inshore patch reef in the Florida Keys, annually from 2012 to 2016. This site experienced warm-water bleaching in 2011, 2014, and 2015. In 2017, we obtained cores of the dominant calcifying coral at this site, *Orbicella faveolata*, to understand how calcification rates were impacted by bleaching and how they affected the reef-wide CaCO_3_ budget. Bleaching depressed *O. faveolata* growth and the decline of this one species led to an overestimation of mean (± std. error) reef-wide CaCO_3_ production by + 0.68 (± 0.167) to + 1.11 (± 0.236) kg m^−2^ year^−1^ when using the static *ReefBudget* coral growth inputs. During non-bleaching years, the *ReefBudget* inputs slightly underestimated gross production by − 0.10 (± 0.022) to − 0.43 (± 0.100) kg m^−2^ year^−1^. Carbonate production declined after the first year of back-to-back bleaching in 2014, but then increased after 2015 to values greater than the initial surveys in 2012. Cheeca Rocks is an outlier in the Caribbean and Florida Keys in terms of coral cover, carbonate production, and abundance of *O. faveolata*, which is threatened under the Endangered Species Act. Given the resilience of this site to repeated bleaching events, it may deserve special management attention.

## Introduction

Much of the ecosystem function of coral reefs is directly linked to their three-dimensional structure (Enochs and Manzello 2012; Graham and Nash 2012). Calcium carbonate (CaCO_3_) production by corals and other calcifiers (e.g. crustose coralline algae, *Halimeda*, bryozoans, etc.) must exceed CaCO_3_ loss due to physical, chemical, and biological erosion for coral reef frameworks to persist (Glynn and Manzello 2015). Climate change and ocean acidification (OA) will reduce the production of CaCO_3_ by corals via mortality from bleaching, as well as depressed coral calcification from sub-lethal thermal stress and decreasing carbonate saturation state (Glynn 1988; Chan and Connolly 2013; Cantin and Lough 2014; Perry and Morgan 2017). Additionally, experimental studies suggest that OA will accelerate coral reef bioerosion and dissolution, possibly leading to net erosion and/or dissolution of reefs globally by the end of the century (Tribollet et al. 2009; Wisshak et al. 2012; Reyes-Nivia et al. 2013; Silbiger et al. 2014; Enochs et al. 2015, 2016a; Eyre et al. 2018).

The CaCO_3_ budget of coral reefs is difficult to measure as many different organisms are involved in the production of CaCO_3_ and its breakdown. Perry et al. (2012) developed a rapid, census-based CaCO_3_ budget monitoring tool for Caribbean coral reefs, termed *ReefBudget*. Using this method, Perry et al. (2013) showed that CaCO_3_ production has declined to 50% below historical averages across the Caribbean, and more than a third of the 101 sites surveyed (37%) were net erosional (Perry et al. 2013). Additionally, by applying the *ReefBudget* method, Enochs et al. (2015) found that 89% of reefs in the Florida Keys were net erosional. In the Florida Keys, net erosion rates ranged from − 0.43 to − 1.6 kg m^−2^ year^−1^, whereas in the wider Caribbean these were − 0.14 to − 1.77 kg m^−2^ year^−1^ (Perry et al. 2013; Enochs et al. 2015). Overall, coral cover has declined by about 80% since the 1970s in the Caribbean and reefs are losing three-dimensional structure (Gardner et al. 2003; Alvarez-Filip et al. 2009). The decline in coral cover is the primary driver of these reefs presently being net erosional, and the loss of coral has been a result of coral bleaching, disease, overfishing, and other local-scale factors like land-based sources of pollution (Williams and Bunkley-Williams 1990; Aronson and Precht 2001; Pandolfi et al. 2005). Given that many Caribbean reefs are at, or are close to CaCO_3_ budget neutral, termed “accretionary stasis”, there is a concern that the persistence of architecturally complex reef framework structures is in jeopardy (Perry et al. 2013). Three-dimensional, architecturally complex reef frameworks are vital to reef ecosystem function, trophodynamics, and the high biodiversity of coral reefs (Enochs and Manzello 2012; Graham and Nash 2012).

The National Oceanic and Atmospheric Administration’s Coral Reef Conservation Program recently instituted the National Coral Reef Monitoring Program (NCRMP) (NOAA Coral Program 2014). The goal of NCRMP is to monitor the status and trends of US reefs, including climate change, OA, and the resultant ecosystem impacts. As part of NCRMP, *ReefBudget* surveys are being conducted at select sites in the Caribbean where high-accuracy and high-precision measurements of carbonate chemistry are taking place. The impetus is to understand how the organisms that drive the CaCO_3_ budget change through time and are impacted from other disturbances such as thermal stress. This will help tease out the potential impacts from OA versus the other stressors that impact the population dynamics of key taxa in the carbonate budget of coral reefs.

**Fig. 1:**
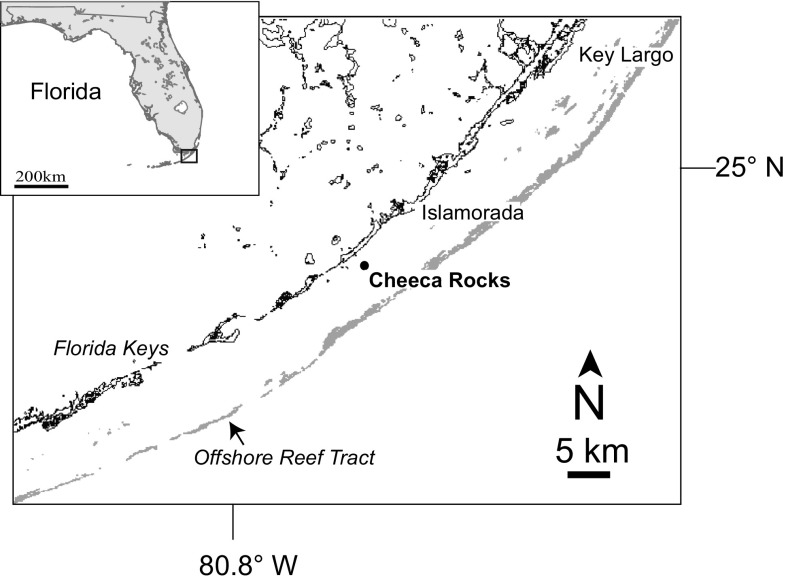
Map showing location of Cheeca Rocks patch reef, Florida Keys, USA. Gray is offshore reef tract

**Fig. 2:**
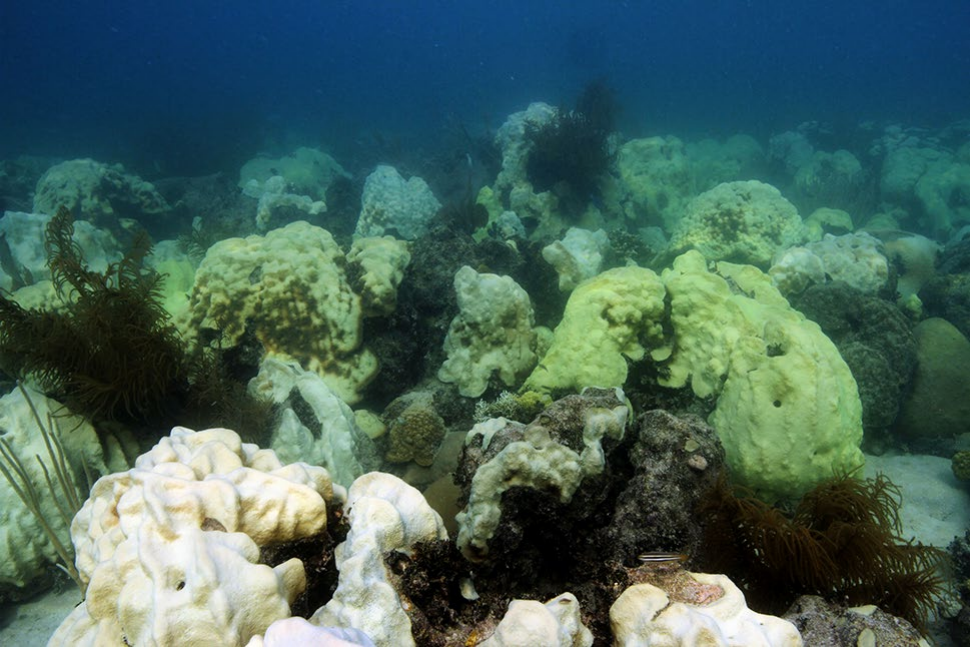
Photograph of coral bleaching at Cheeca Rocks in September 2014. This image represents the high abundance of *Orbicella faveolata* at this site, as well as the severity of bleaching in 2014

Annual CaCO_3_ budget surveys have been conducted at Cheeca Rocks, an inshore patch reef in the Florida Keys, since 2012 (Fig. 1). In the summers of 2014 and 2015, mass coral bleaching events impacted the entire Florida Reef Tract (Fig. 2), the sixth and seventh Florida Keys-wide event since 1987. A localized bleaching event also took place at Cheeca Rocks in the summer of 2011 due to anomalously warm waters impacting inshore reef environments (Manzello et al. 2015a, b). This paper reports on the year-to-year variability in CaCO_3_ cycling from 2012 to 2016. Particular focus is given to the impacts of bleaching on CaCO_3_ production in 2011, 2014 and 2015 measured from coral cores of the dominant carbonate-producing coral species, *Orbicella faveolata*, that were collected in 2017. We compare and contrast the reef-wide carbonate production estimates using the default *ReefBudget* inputs for coral growth to those using the locally measured calcification rates for the dominant carbonate producer. These findings highlight the benefits of using local rate data where possible, as proposed in the original *ReefBudget* methodology.

## Methods

In May 2012, six permanent 10-m transects were established by hammering rebar into the substrate and have been surveyed annually at Cheeca Rocks (24.8977N, 80.6182W), which is a shallow (depth range 2–6 m) inshore patch reef in the Florida Keys, adjacent to Islamorada (Fig. 1). Carbonate budget surveys followed the *ReefBudget* protocols described by Perry et al. (2012). In brief, a chain transect survey is conducted along each 10-m linear transect whereby a flexible chain is laid upon the reef and all the organisms and substrate types underlying the chain are counted. Corals are identified to species, while other major taxa are categorized based on functional group, including crustose coralline algae, *Halimeda*, and sponge.

Three different types of surveys document the prevalence of bioeroding taxa. First, the surface area of clionaid sponge cover is determined for 0.5 m on either side of each 10-m linear transect using a quadrat. Second, the test diameter of each urchin species encountered within 1 m of either side of each 10-m transect is measured using hand-held calipers. Lastly, 30-m by 4-m belt transects (*n* = 10) are conducted to measure parrotfish abundances across the six sites. Parrotfish are identified to species, phase, and size class according to the *ReefBudget* methodology (Perry et al. 2012).

The CaCO_3_ budget model at Cheeca Rocks was first parameterized using the default *ReefBudget* inputs, and then by inputting site-specific coral growth data from 5-cm-diameter × 10-cm-long cores of *Orbicella faveolata* (*n* = 6, 2–3 m water depth) that were collected using a pneumatic hand-drill in May 2017. Six additional cores for which data were published in Manzello et al. (2015a) provided additional growth data from 2012 to 2014. As such, the growth data from 2012 and 2013 represent the means of 12 cores, 2014 represents 8 cores, and 2015–2016 the recent six cores. The default *ReefBudget* inputs represent averages from a meta-analysis of multiple growth studies for each species of coral and bioeroder (Perry et al. 2012). Finally, the CaCO_3_ budget model was also parameterized by assuming that all coral species at this site had the same growth dynamics as *O. faveolata* relative to the default *ReefBudget* values. For example, calcification of *O. faveolata* was 81.8% of the *ReefBudget* value in 2012 (Table 1), so we assumed every coral species’ calcification rates were 81.8% of the *ReefBudget* input in 2012.

**Table 1:** Mean (± SEM) linear extension (cm year^−1^), skeletal density (g cm^−3^), and calcification (g cm^−2^ year^−1^) of *Orbicella faveolata* at Cheeca Rocks from 2012 to 2016

Year	Linear extension	Skeletal density	Calcification
2012	0.72 (0.077)	1.31 (0.045)	0.96 (0.122)
2013	1.07 (0.089)	1.24 (0.050)	1.30 (0.095)
2014	1.03 (0.058)	1.17 (0.047)	1.19 (0.076)
2015	0.62 (0.052)	1.35 (0.042)	0.85 (0.086)
2016	0.67 (0.080)	1.32 (0.082)	0.86 (0.064)

Year	Extension %	Density (%)	Calcification (%)
2012	85.1	94.1	81.8
2013	126.8	89.2	111.1
2014	121.9	84.0	102.0
2015	73.9	97.2	72.3
2016	79.8	94.8	73.4

Values also expressed as a percentage of the default *ReefBudget* value for this species (*ReefBudget* values for *O. faveolata:* Ext. = 0.842 cm year^−1^; density = 1.39 g cm^−3^; calcification 1.17 g cm^−2^ year^−1^)

*SEM* standard error of the mean

Coral cores were analyzed using a Siemens Somatom Volume Zoom spiral computerized tomography (CT) Scanner at 0.1 mm resolution. Density measurements were made along the growth axis in the CT images using Amira software (FEI Visualization Sciences Group, Massachusetts, USA). Density (g cm^−3^) was determined from grayscale values by linear regression of coral standards of known density as previously described (Groves et al. 2018). Linear extension (cm year^−1^) was determined by measuring the distance between annually repeating high-density bands using the Coral X-radiograph Densitometry System (CoralXDS) (Helmle et al. 2002). Calcification rates (g cm^−2^ year^−1^) were calculated as the product of density and linear extension. For simplicity in reporting, we pooled *O. faveolata* and *Orbicella annularis* into one category labelled “*Orbicella annularis* species complex” because for some of the smaller colonies it was not possible to differentiate between these two species. *O. faveolata* accounted for 88–96% of the *O. annularis* spp. encountered from 2012 to 2016, while *O. annularis* made up the remainder. The *O. faveolata* growth data from the cores were only input into the carbonate budget model for the confirmed *O. faveolata* colonies and not any other *O. annularis* species.

The carbonate budget surveys took place in mid-summer, generally July, thus it was assumed the coral growth rates inputted into the model were integrated over the past year. For example, for surveys in 2014, the coral growth data input integrated the growth measured from the time of high-density band formation in 2013 to high-density band formation in 2014. High-density band formation in this species occurs in the late summer (Hudson et al. 1976). The impacts of bleaching in 2014 and 2015 thus manifested in the 2015 and 2016 carbonate budget output. Statistical analyses were performed using Sigma-Plot 12.

## Results

**Fig. 3:**
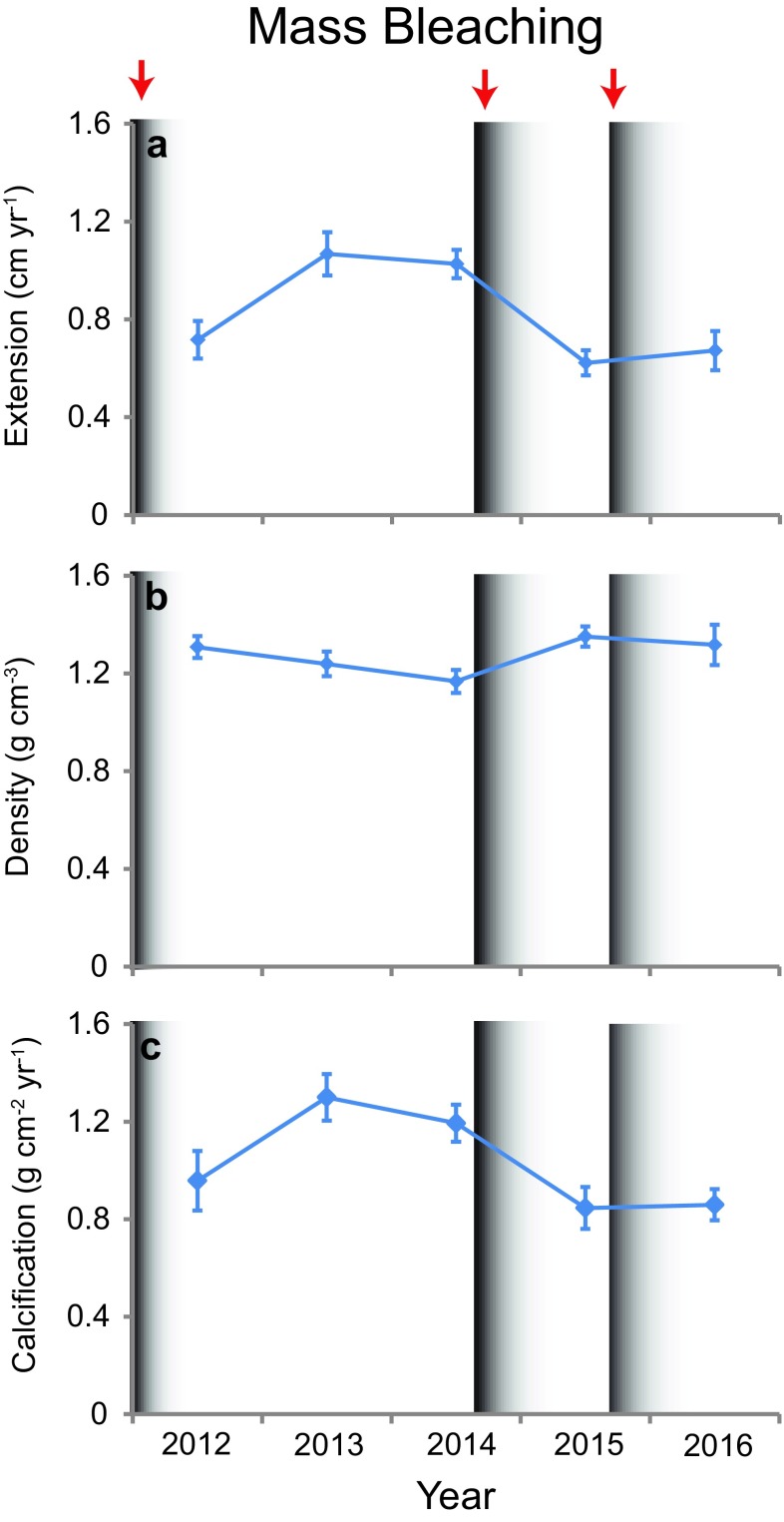
Mean **a** linear extension, **b** density, and **c** calcification rate per year from 2012 to 2016 at Cheeca Rocks. Error bars are std. error of mean. Bleaching events in 2011, 2014, and 2015 are indicated by red arrows and shaded black bars

The extension rates of *O. faveolata* were significantly impacted by warm-water bleaching in 2011, 2014, and 2015 (Fig. 3, Tables 1, 2). Cheeca Rocks experienced 7.7 degree heating weeks (DHWs) in 2014 and 9.5 in 2015 (Gintert et al. 2018). DHWs are a measure of the magnitude and duration of sea temperatures ≥ 1 °C above the maximum monthly mean temperature and are the most often used metric of thermal stress for coral reefs (Liu et al. 2006). In situ temperature data from Cheeca Rocks are not available for 2011, but for nearby Molasses Reef, DHWs were 0.9 less in 2011 relative to 2014. Cheeca Rocks and Molasses Reef have similar patterns in sea temperatures, so it is likely that the differences in magnitude of thermal stress between the three bleaching years were similar (Gintert et al. 2018). There was a significant depression in calcification in 2015 due to bleaching in 2014, but the 2016 values were not significantly different from the other years despite being noticeably depressed from bleaching in 2015 (Fig. 3). Density slightly increased after bleaching, but was not significant.

**Table 2:** Results of repeated measures ANOVA for CaCO_3_ budget parameters by year from 2012 to 2016

	Calcification rate input		
	*ReefBudget*	*O. faveolata* input	All corals
Variable			
Reef-wide CaCO_3_ production/bioerosion			
Gross	ns	ns	ns
Net	ns	ns	ns
*O. annularis* spp.	ns	*F* = 3.2, *P* < 0.05 pairwise ns	*F* = 3.4, *P* < 0.05 pairwise ns
Coral cover (%)	ns	n/a	n/a
*Orbicella faveolata* growth data			
Extension	*F* = 6.6, *P* < 0.001 2013 > 2012, 2015, 2016 2014 > 2015	n/a	n/a
Density	ns	n/a	n/a
Calcification	*F* = 3.7, *P* < 0.05 2013 > 2015	n/a	n/a
Bioerosion			
Total	*F* = 3.0, *P* < 0.05, 2014 > 2013	n/a	n/a
Bioerosion by taxa			
Urchin*	*χ*^2^ = 10.6, *P* < 0.05 2016 < 2012	n/a	n/a
Parrotfish	*F* = 4.8, *P* < 0.01 2014 > 2012, 2013	n/a	n/a
Clionaid sponges	ns	n/a	n/a
Microborers	*F *= 4.1, *P* < 0.05 2016 > 2013, 2014	n/a	n/a

Tukey pairwise comparison used when ANOVA indicated significant differences between years

*ns* not significant, *n/a* not applicable

*Data not normal, repeated measures ANOVA on ranks (Friedman test) used

**Fig. 4:**
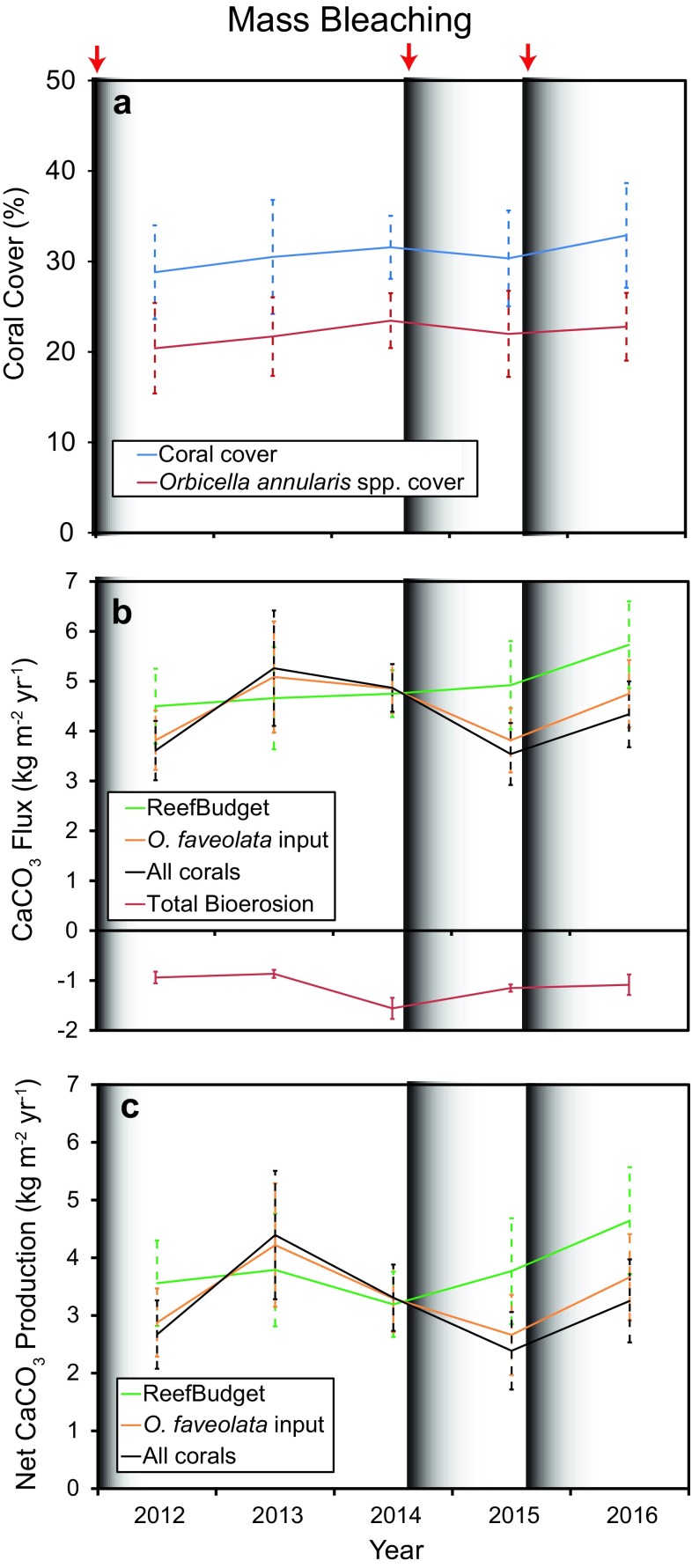
Coral cover and CaCO_3_ flux at Cheeca Rocks from 2012 to 2016. **a** Percent cover of all corals (blue) and *Orbicella annularis* species complex (red). **b** Gross CaCO_3_ production and bioerosion (red line). **c** Net CaCO_3_ production. CaCO_3_ production estimates using *ReefBudget* input are in green, whereas production data generated with locally measured *Orbicella faveolata* calcification data are shown by an orange line. The black line shows production values when the same relative offset measured for the *O. faveolata* data versus the default *ReefBudget* value was applied to all coral species. Values are means ± std. error of the mean

Coral cover increased 4.5% from 2012 to 2016, but this was not significant (Fig. 4, Tables 2, 3). The *O. annularis* spp. complex dominated the coral community, making up 69.3–73.6% of total cover. *Siderastrea siderea, Porites astreoides*, and *Colpophyllia natans* were the next three species with the highest cover, respectively. *O. faveolata* was the dominant species, accounting for > 88% of the *O. annularis* spp. measured. Both total coral cover and cover of the *O. annularis* spp. declined by 1.3% after the 2014 bleaching event (Fig. 4). Cover of *O. annularis* spp. was 0.5% lower in 2016 (22.8%) than it was prior to bleaching (23.3% in 2014), but still higher than it was at the beginning of monitoring in 2012 (20.2%).

**Table 3:** CaCO_3_ production and bioerosion by year from 2012 to 2016

CaCO_3_ budget metrics	2012	2013	2014	2015	2016
Coral cover (%)	28.4 (4.69)	29.6 (6.31)	31.6 (3.48)	30.3 (5.29)	32.9 (5.79)
*O. annularis* spp. cover (%)	20.2 (4.45)	21.1 (4.35)	23.3 (3.03)	22.0 (4.76)	22.8 (3.75)
*O. annularis* spp. % of total cover	69.5 (8.47)	69.6 (7.09)	73.5 (7.04)	66.0 (9.86)	70.0 (5.57)
Gross CaCO_3_ production					
*ReefBudget* inputs					
Gross production	4.50 (0.746)	4.66 (1.022)	4.75 (0.467)	4.92 (0.869)	5.73 (0.873)
*O. annularis* spp. production	3.51 (0.764)	3.55 (0.721)	3.86 (0.501)	3.91 (0.842)	4.37 (0.723)
*O. annularis* spp. % of total prod.	75.3 (7.24)	75.6 (5.98)	80.0 (5.53)	72.2 (9.85)	75.1 (4.49)
Local *O. faveolata* data input					
Gross production	3.82 (0.595)	5.09 (1.114)	4.85 (0.486)	3.81 (0.643)	4.75 (0.677)
*O. annularis* spp. production	2.83 (0.599)	3.98 (0.819)	3.96 (0.521)	2.81 (0.605)	3.39 (0.450)
*O. annularis* spp. % of total prod.	72.3 (7.77)	77.1 (5.86)	80.3 (5.48)	67.0 (10.30)	72.3 (5.21)
All corals modeled					
Gross production	3.61 (0.595)	5.26 (1.157)	4.89 (0.478)	3.54 (0.621)	4.34 (0.660)
*O. annularis* spp. production	2.81 (0.612)	4.01 (0.815)	3.95 (0.513)	2.81 (0.605)	3.30 (0.509)
*O. annularis* spp. % of total prod.	74.9 (7.28)	75.7 (5.94)	80.0 (5.53)	72.1 (9.94)	76.0 (5.04)
Total bioerosion	− 0.94 (0.117)	− 0.87 (0.081)	− 1.56 (0.211)	− 1.15 (0.073)	− 1.08 (0.206)
Bioerosion by taxa					
Urchin	− 0.18 (0.114)	− 0.07 (0.054)	− 0.12 (0.105)	− 0.06 (0.045)	− 0.01 (0.006)
Parrotfish	− 0.50 (0.087)	− 0.56 (0.120)	− 1.21 (0.192)	− 0.83 (0.052)	− 0.80 (0.202)
Clionaid sponges	− 0.004 (0.0028)	− 0.005 (0.0030)	− 0.005 (0.0040)	− 0.001 (0.0005)	− 0.002 (0.0016)
Microbioerosion	− 0.26 (0.017)	− 0.23 (0.019)	− 0.22 (0.021)	− 0.26 (0.027)	− 0.28 (0.030)
Net CaCO_3_ production					
*ReefBudget*	3.56 (0.740)	3.79 (0.978)	3.19 (0.565)	3.77 (0.917)	4.64 (0.926)
Local *O. faveolata* input	2.88 (0.591)	4.22 (1.070)	3.29 (0.584)	2.66 (0.696)	3.66 (0.748)
All corals modeled	2.67 (0.593)	4.39 (1.112)	3.31 (0.575)	2.39 (0.670)	3.25 (0.719)

Values are means (± std. error of the mean). Reef-wide production and bioerosion values are kg CaCO_3_ m^−2^ year^−1^

Gross CaCO_3_ production increased every year when the default *ReefBudget* inputs for coral calcification were used, ranging from 4.50 ± 0.746 kg m^−2^ year^−1^ (mean ± std. error) in 2012 to 5.73 ± 0.873 kg m^−2^ year^−1^ in 2016 (Table 3, Fig. 4). *O. annularis* spp. were responsible for 72–80% of total production in any given year. Using the *ReefBudget* inputs, gross production increased by 0.17 kg m^−2^ year^−1^ in 2015 after bleaching in 2014 despite a decline in coral cover. This was because of changes in the abundances of multiple taxa (Table 4). Despite declines in some species (*O. annularis, Porites astreoides, Porites porites*) there were increases in others (*O. faveolata, Siderastrea siderea, Stephanocoenia intersepta*) that led to the slight increase in CaCO_3_ production.

**Table 4:** Difference in CaCO_3_ production (kg m^−2^ year^−1^) before and after bleaching in 2014 when using default *ReefBudget* calcification inputs

Taxa	ΔCaCO_3_ production
*Orbicella faveolata*	+ 0.55
Crustose coralline algae (CCA)	+ 0.02
*Colpophyllia natans*	+ 0.02
*Diploria labyrinthiformis*	+ 0.008
*Siderastrea radians*	+ 0.007
*Siderastrea siderea*	+ 0.086
*Stephanocoenia intersepta*	+ 0.077
*Orbicella annularis*	− 0.49
*Pseudodiploria strigosa*	− 0.003
Macroalgae covered with CCA	− 0.004
*Montastraea cavernosa*	− 0.002
*Porites astreoides*	− 0.073
*Porites porites*	− 0.021
Net change	+ 0.17

**Fig. 5:**
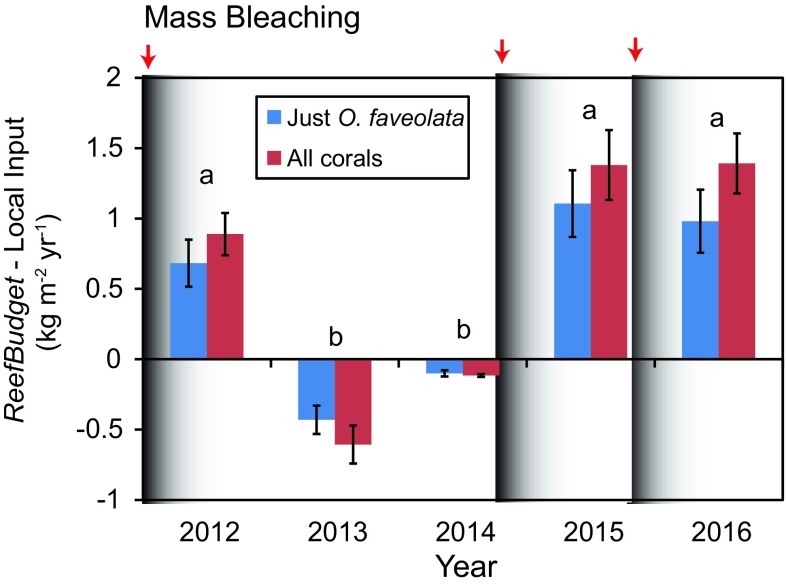
Annual difference in gross carbonate production estimated by *ReefBudget* inputs and locally derived calcification data. Blue is when only *O. faveolata* data were used, whereas red is when all coral species were modeled. Values are means ± std. error of the mean. Different letters denote significantly different years as indicated by Tukey post hoc comparisons

When the locally measured calcification rates of *O. faveolata* were input into the model, gross and net carbonate production were more variable and followed a similar pattern to the calcification rates measured in the cores (Fig. 4). There were no significant differences by year for gross or net carbonate production when the core data were used despite the notable declines following bleaching (Table 2). However, there was a significant effect by year when the differences between the gross production outputs using the *ReefBudget* and the locally measured *O. faveolata* data were examined (Repeated Measures Analysis of Variance, *F* = 19.1, *P* < 0.001) (Fig. 5). The bleaching-impacted years of 2012, 2015 and 2016 were significantly different than those years at least 1 year removed from a bleaching event (2013, 2014) (Tukey post hoc tests, *P* < 0.05). When *O. faveolata* calcification rates were impacted by bleaching, the *ReefBudget* inputs overestimated mean (± std. error of mean) rates of gross reef calcification by + 0.68 (± 0.167) in 2012 to + 1.11 (± 0.236) kg m^−2^ year^−1^ in 2015 (Fig. 5). During non-bleaching years, the *ReefBudget* inputs slightly underestimated (relative to the site-parameterized calcification model) gross production by − 0.10 (± 0.022) to − 0.43 (± 0.100) kg m^−2^ year^−1^. Not surprisingly, the overestimations of gross production by the *ReefBudget* inputs were greater when it was assumed that all the coral species at Cheeca Rocks grew in the same way as *O. faveolata* relative to the *ReefBudget* inputs (*F* = 30.7, *P* < 0.01) (Fig. 5).

**Fig. 6:**
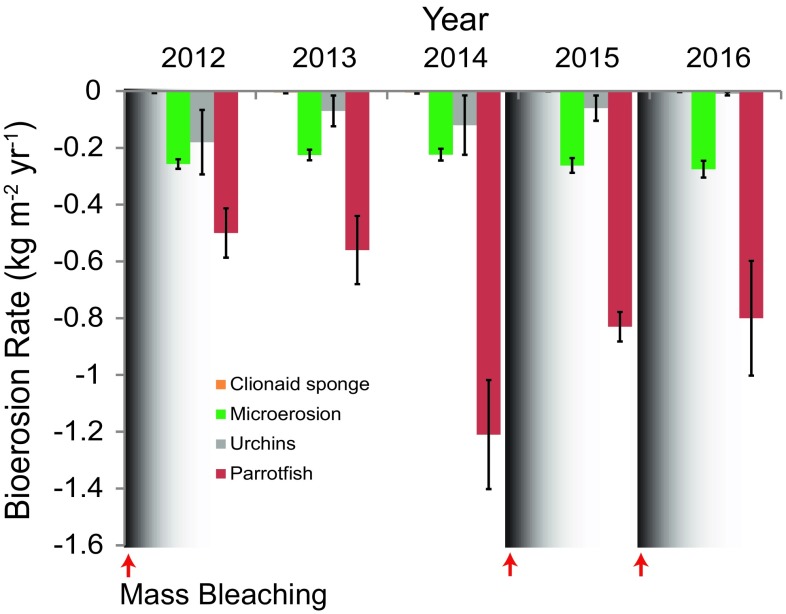
Bioerosion rate by taxa from 2012 to 2016. Values are means ± std. error of the mean

Parrotfish accounted for 53.2–77.6% of the total bioerosion at Cheeca Rocks, ranging from − 0.50 (± 0.087) in 2012 to − 1.21 (± 0.192) kg m^−2^ year^−1^in 2014 (Fig. 6, Table 3). Parrotfish bioerosion was significantly greater in 2014 than the prior 2 years with rates more than doubling in a single year (Tables 2, 3). Parrotfish were mainly juvenile striped parrotfish, *Scarus iserti*. Microbioerosion was the second largest contributor to total bioerosion, followed by urchins in the genus *Echinometra* (Fig. 6). Clionaid sponge bioerosion was negligible. In 2016, after two consecutive years of bleaching, urchin bioerosion was the lowest since 2012, whereas microbioerosion was the highest (Table 3).

## Discussion

Sublethal bleaching in 2011, 2014, and 2015 significantly impacted the extension and calcification rates of the dominant CaCO_3_ producer, *O. faveolata* at Cheeca Rocks, which in turn led to an overestimation of gross CaCO_3_ production during these bleaching-impacted years when the default coral growth rates in *ReefBudget* were used. This is not surprising given that the *ReefBudget* calcification inputs represent an average of growth rates for each species during unstressed conditions (Perry et al. 2012). Furthermore, coral bleaching is well known to depress coral calcification and linear extension (Cantin and Lough 2014), as has been previously shown at Cheeca Rocks (e.g., Manzello et al. 2015a, b). These data confirm the previously reported low extension and calcification after the 2011 bleaching event followed by a quick recovery in the following year (Manzello et al. 2015a). During non-bleaching years, the *ReefBudget* inputs tended to underestimate gross production for this site because growth rates at Cheeca Rocks for *O. faveolata* were greater than the default values (Table 1). Overall, the *ReefBudget* default inputs yielded gross production data that were generally in good agreement with the production rates estimated with the actual growth data when calcification of the dominant calcifier was not impaired by bleaching.

Cheeca Rocks exhibited resilience in CaCO_3_ production with back-to-back coral bleaching events in 2014 and 2015, despite these being the two hottest years on record (Manzello 2015; Gintert et al. 2018). Coral cover unexpectedly increased in our six transects from 2012 to 2016, despite a slight decline after the 2014 bleaching. In a companion study utilizing landscape mosaic photographic imagery of these same sites from 2012 to 2016, but incorporating a larger area per site (10 m × 10 m), a similar resilience to bleaching was documented (Gintert et al. 2018). The Gintert et al. study did, however, document a 3.7% decline in coral cover as a result of the two bleaching events (from 29.2% in 2014 to 25.5% in 2016), but bleaching prevalence, severity, and mortality were lower during the second year of bleaching despite there being greater thermal stress in 2015. The disparity in coral cover changes over the same time frame between these two studies may be because the CaCO_3_ production data presented herein are parameterized by approximately 60 total linear meters of reef per year, whereas the other study surveyed 600 m^2^ of reef per year. Despite these differences, both studies indicate that the 2014 and 2015 bleaching events did not have an overly severe impact on the coral community at Cheeca Rocks.

**Fig. 7:**
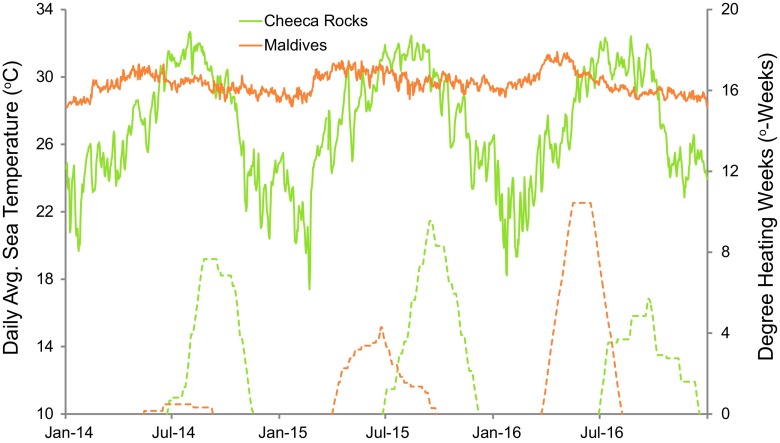
Daily average sea temperatures and degree heating weeks from Cheeca Rocks and the Maldives. Data from Cheeca Rocks are in situ temperature data from Gintert et al. (2018), whereas the Maldives data are from NOAA’s Coral Reef Watch (Accessed 28 Feb 2018: https://coralreefwatch.noaa.gov/vs/index.php)

This contrasts greatly with other sites that experienced high degrees of coral mortality and consequently large declines in CaCO_3_ production after bleaching (e.g., Glynn 1988; Perry and Morgan 2017). A recent study using the *ReefBudget* methodology in the Maldives found that a single bleaching event in 2016 led to a 75% decline in coral cover and 78% decline in gross carbonate production in 8 months (Perry and Morgan 2017). One explanation for these differences is that the Maldives experienced slightly greater thermal stress with a maximum of 10.4 degree heating weeks (DHW, a metric for thermal stress dosage, Liu et al. 2006) versus a maximum of 9.5 DHW at Cheeca Rocks (Fig. 7). It seems unlikely, though, that an increase of as little as < 1 DHW would be the sole factor for such different outcomes, especially given that Cheeca Rocks experienced two consecutive bleaching events.

High mortality of corals in the genus *Acropora* (91% decline in cover) was the primary driver of the large decline in gross CaCO_3_ production in the Maldives, as they accounted for 52–62% of all coral carbonate production before bleaching (Perry and Morgan 2017). *Acropora* in Florida, much like the wider Caribbean, have undergone drastic population declines since the 1980s and were never abundant at sites like Cheeca Rocks, given the suboptimal environmental conditions (Shinn 1966; Miller et al. 2002). Cheeca Rocks is a small inshore patch reef, approximately 2 km from shore and 20 ha in areal extent, which experiences high and low temperature extremes, as well as increased sedimentation, turbidity and nutrients relative to offshore reefs (Lirman and Fong 2007). Sea temperatures in the summer are generally 1 °C warmer on the inshore sites, while in the winter they are usually > 1 °C cooler (Manzello et al. 2012, 2015b). For further information on specific values of sedimentation, turbidity and nutrients, see Lirman and Fong (2007). The absence of the most thermally sensitive *Acropora* species at Cheeca Rocks is likely an additional reason for the muted response relative to the Maldives. Branching corals, like the acroporidae, tend to be more thermally sensitive than massive corals (Loya et al. 2001). Coral reefs in marginal environments like those at Cheeca Rocks have been more resilient to thermal stress events around the globe (van Woesik et al. 2012; Guest et al. 2016; Morgan et al. 2017; Gintert et al. 2018). It is unclear if marginal coral communities will continue to be resilient under greater or more frequent thermal stress, which is expected with climate change.

Bioerosion at Cheeca Rocks was dominated by parrotfish, which agrees with the other studies on Caribbean Reefs using this method (Perry et al. 2012, 2013). The decline in net CaCO_3_ production in the Maldives was even greater (− 157.5%) than the decline in gross production because parrotfish bioerosion increased 139.5% after bleaching (Perry and Morgan 2017). There was no obvious linkage between parrotfish abundances and bleaching at Cheeca Rocks, which is probably because the lack of coral mortality did not create new grazing space. Before the bleaching event, parrotfish abundance and bioerosion more than doubled from 2013 to 2014, but declined thereafter (Fig. 6). This was likely due to a recruitment pulse as there was a large increase in the numbers of small, juvenile striped parrotfish (*S. iserti*) in 2014 that then declined the following year. Microbioerosion played the second biggest role in bioerosion at Cheeca Rocks. Although microbioerosion significantly increased after the back-to-back bleaching events in 2014 and 2015, the increase was only + 0.05 to 0.06 kg m^−2^ year^−1^ (Table 3). The magnitude of this change is of limited importance to the overall carbonate budget. Urchin abundances and bioerosion were highest in the first year of monitoring and declined to very low values after the two bleaching events, but it is not clear if this is related to the bleaching events or if this just represents normal urchin population fluctuations. This is an opposite pattern to what occurred in the eastern Pacific after bleaching when urchin bioerosion became the most important factor in the carbonate budget on reefs in both Panamá and the Galápagos Islands (Glynn 1988, 1990).

Thermal stress events can negatively impact many coral reef organisms, in addition to zooxanthellate corals, including organisms with and without algal symbionts (Williams and Bunkley-Williams 1990). Thus, it is important to consider that the rates of bioerosion and calcification from organisms other than corals are also dynamic and are likely responding to temperature anomalies, as well as to increasing OA (Kennedy et al. 2013). Microbioerosion increases with temperature, OA, as well as during coral bleaching due to increased light penetration through bleached coral tissues that leads to increases in biomass of endolithic algae (Fine and Loya 2002; Tribollet et al. 2009; Reyes-Nivia et al. 2013; Enochs et al. 2016b). Clionaid sponges respond positively to OA and temperature, but zooxanthellate species are sensitive to thermal stress (Wisshak et al. 2012; Fang et al. 2014; Enochs et al. 2015a). Urchins are negatively impacted by OA and thermal stress (Uthicke et al. 2014), although the genera at Cheeca Rocks (*Echinometra*) showed the greatest sensitivity to OA at low temperatures (Courtney et al. 2013). Finally, parrotfish grazing increases with temperature (Smith 2008). For a holistic understanding of CaCO_3_ budgets, monitoring should incorporate census-based approaches like *ReefBudget,* routine species-specific coral calcification monitoring, as well as hydrochemical approaches (Courtney et al. 2016).

Cheeca Rocks is an outlier among Caribbean reefs and, most especially, among the degraded coral reefs of the Florida Keys (Ruzicka et al. 2013). The higher coral cover on the inshore reefs has been hypothesized to be due to increased resistance and/or resilience of local corals to elevated temperatures and bleaching (Kenkel et al. 2013; Kenkel and Matz 2016). It is unlikely that direct human impacts are involved as the inshore sites are closest to human population centers and land-based sources of pollution. The mechanism for this increased bleaching tolerance is unclear, but may be related to coral host and/or symbiont adaptation and/or acclimatization to high and variable temperatures, higher turbidity leading to increased feeding, and/or bleaching-mitigating environmental factors (lower light) (Lirman and Fong 2007). The minimum coral cover we measured (28.4% in 2012) was higher than 91% of the 101 individual transects measured across the Bahamas, Cayman Islands, Belize, and Bonaire by Perry et al. (2013). The lowest mean gross carbonate production at Cheeca Rocks using both the default *ReefBudget* and local inputs during non-bleaching years was higher than 76% of the transects occupied by Perry et al. (2013). Even the minimum gross production value using the bleaching-impacted local values were still greater than 64% of the Perry et al. (2013) sites. The gross and net rates of production at Cheeca Rocks are the highest measured to date for Florida (Enochs et al. 2015). The *O. annularis* spp. complex made up 67–80.3% of the carbonate production and 66–73.5% of the total coral cover at Cheeca Rocks from 2012 to 2016. These species are listed as threatened under the Endangered Species Act (ESA) (Brainard et al. 2011). Despite 2014 and 2015 being the two warmest years on record in the Florida Keys, these two bleaching events did not have a clear, long-term impact on CaCO_3_ production at Cheeca Rocks. This site may be a refuge for the ESA-listed *O. annularis* spp. and, in particular, *O. faveolata,* and deserving of special protection by the Florida Keys National Marine Sanctuary.
